# Community Case Study: Stack Up’s Overwatch Program, an Online Suicide Prevention and Peer Support Program for Video Gamers

**DOI:** 10.3389/fpsyg.2021.575224

**Published:** 2021-03-11

**Authors:** Michelle Colder Carras, Mathew Bergendahl, Alain B. Labrique

**Affiliations:** ^1^Department of International Health, Johns Hopkins University Global mHealth Initiative, Johns Hopkins Bloomberg School of Public Health, Baltimore, MD, United States; ^2^Stack Up, Sylmar, CA, United States

**Keywords:** suicide prevention, video games, internet, veterans, mental health services research, online communities, peer support, Discord

## Abstract

Traditional mental health services are often not enough to meet the needs of people at risk for suicide, especially in populations where help-seeking is stigmatized. Stack Up, a non-profit veteran organization whose goal is to use video games to bring veterans together, recognized a need in its gaming-focused online community and created the Overwatch Program. This suicide prevention and crisis intervention program is delivered entirely through the Internet by trained community members through Discord text and voice chat. By combining aspects of virtual gaming communities, veteran mental health, and community-based peer support, this program provides an innovative format for implementing crisis intervention and mental health support programs. We describe here the context and features of the program, an ongoing evaluation project, and lessons learned.

## Introduction

Mental disorders are prevalent and potentially disabling conditions worldwide, and suicide prevention specifically remains an urgent priority for mental health research and practice (National Institute of Mental Health [NIMH], 2020). Suicide rates continue to rise in the United States, despite progress in reducing mortality from other diseases (Case and Deaton, 2015; Stone, 2018). Veterans have been identified as a vulnerable population with a higher risk of suicide (Ashrafioun et al., 2016) and large unmet mental health need (National Institute of Mental Health [NIMH], 2020). Risk factors associated with suicide, such as financial problems, loneliness/social isolation, and anxiety, may be worsened by the pandemic and exacerbate pre-existing mental or behavioral health conditions for veterans (Armstrong, 2020; Gunnell et al., 2020). Many veterans experiencing psychological distress, mental or behavioral health problems, or significant life stressors are reluctant to seek help (Spelman et al., 2012; Quartana et al., 2014). For example, a recent survey of over 4,000 veterans estimates that 41% of recent veterans, or about 1.7 million veterans, have mental health need based on well-validated mental health screeners (National Academies of Sciences, Engineering, and Medicine [NASEM], 2018). These findings also show that over half of those with potential unmet need have not sought mental health treatment, either in the Department of Veterans Affairs (VA) or elsewhere.

Barriers to receiving mental health services come from several areas, as outlined in a recent research brief summarizing the results of several studies of veteran mental health (RAND, 2019). Shortages in the mental health workforce within the VA mean that appointments may not be easily available, and there is wide variability in the delivery of evidence-based treatments within this system. Veterans express concern about the effectiveness of mental health treatment, including side effects of medication, but also express fears that their career might be affected by seeking treatment, or that looking for help is a sign of weakness. Providing a wide range of choices for mental health support and treatment makes it easier to address needs and overcome barriers (Hoge, 2016). Telemental health has been specifically recommended as a way to expand the provision of evidence-based care (RAND, 2019). Peer support (in this case, care delivered by veterans with the lived experience of mental or behavioral health problems) is another form of care provided to veterans and is a mandatory offering in the VA system (Department of Veterans Affairs [DVA] and Veterans Health Administration [VHA], 2008). Peer support is generally highly regarded by veterans and clinical leadership (Chinman et al., 2012). Although peer specialist services have promising effects on mental health symptoms for some groups of veterans who engage highly with them (e.g., those with severe mental health problems), broad implementation has been delayed by organizational uncertainty about the role and by inadequate funding (Chinman et al., 2017).

Identifying and assisting people at risk for suicide, responding effectively to crisis, increasing help-seeking, and fostering connectedness are among the recommended strategies for crisis intervention (Suicide Prevention Resource Center [SPRC], 2020). According to Joiner’s interpersonal–psychological theory of suicidal behavior Joiner et al. (2009), social isolation or a feeling of alienation or lack of connectedness (low belongingness) is a robust predictor of suicidal behavior across various populations. Low belongingness is a painful emotional state characterized by not feeling connected to or cared about by others and has been a target of suicide prevention interventions (e.g., Motto and Bostrom, 2001). However, finding scalable ways to support connectedness and mental health and intervene during times of crisis are still a challenge. In the crisis intervention space, anonymous phone-based hotlines have been standard, but with increasing modes of communication, hotlines are branching out into text chat. For example, the Veterans Crisis Line now offers text messaging, online chat, and voice-based crisis intervention for veterans. In responding to over 629,000 calls (Veterans Health Administration [VHA], 2019), this service provides a crucial service to veterans in crisis. However, the one-to-one format of these interventions does not allow for a sense of belonging to a community that is protective against suicide (Chu et al., 2017).

The video gaming community has taken an alternative approach to mental health support and crisis intervention by focusing specifically on belonging. Efforts to address mental health stigma, promote good mental health, and provide mental health support have been ongoing for years through grassroots (i.e., community-developed) and non-profit organizations (History-Rise Above The Disorder, 2020; Take This, 2020; Virtual Ability, 2020) and others. Research supports the match between hedonic (mood- or happiness-based) and eudemonic (autonomy, functioning, and meaning-based) aspects of gaming as well as of mental health. Games are fun, engaging, and often challenging. These hedonic aspects of games and gaming can contribute to wellbeing by promoting positive mood, by providing relief from everyday life stress, or by promoting the rewarding mental state of “flow” (Csikszentmihalyi, 1990; Bowman and Tamborini, 2012; Coulombe et al., 2016; Reinecke and Eden, 2017; Rigby and Ryan, 2017). These positive mood states allow for mindfulness-like decentering (Reinecke and Eden, 2017) that can offer distraction from psychological symptoms and stressors, and this distraction has been linked with adaptive coping aspects of mental health recovery (Colder Carras et al., 2018b). In addition, games offer opportunities for social connection and support, competence, autonomy, and meaningful roles, all important eudemonic aspects of mental health. The anonymity of online settings and the teamwork necessary to overcome challenges promote friendship formation (Kowert, 2014), whereas the ability to control aspects of gameplay fosters a sense of competence and autonomy (Rigby and Ryan, 2017). Leading a guild or team, starting an in-game business, or even performing a specific function in a temporary group (e.g., the healer or tank) are all ways gamers benefit from the eudemonic opportunities games provide (Yee, 2006; Rigby and Ryan, 2017; Rogers et al., 2017). Together, these factors promote mental health, resilience, and participation in the community and society. Programs that promote mental health are a good fit for the gaming community, given that games themselves can meet both hedonic and eudemonic needs. Previous work with the veteran gaming community has shown that playing video games helps veteran gamers in treatment for mental health problems cope with and recover from mental health issues while providing ways to connect and form meaningful bonds with others who play games (Colder Carras et al., 2018b).

Founded in 2015, Stack Up’s mission is to fight the effects of depression, combat injuries, and post-traumatic stress by bringing veterans together through the “shared language” of video games. A “stack” is a slang term for a formation used in military or law enforcement, when an assault team forms up single file along the entrance or doorway to a room where they believe a threat is located. For Stack Up, the stack represents a strong community of friends, family, brothers and sisters in arms, and supporters, all coming together for the common mission of supporting veterans through shared online gaming experiences.

By providing mental health support through the video game community, Stack Up seeks to truly meet veterans where they are—in games and online. Stack Up leadership identified a need in the veteran and military gaming community and responded to it as grassroots organizations do. Although there are no known representative sample studies describing the prevalence or use of gaming in veterans, one study of young adults (18–40 years old) recruited samples from Facebook, targeting individuals who expressed interest in military or veteran pages and military-themed games. Of those who clicked on recruitment ads, were veterans, and met the eligibility criteria; between half and three-quarters played video games at least 1 h per week (Grant et al., 2018). Whether a majority of young adult veterans play games or not (i.e., given the bias inherent in samples recruited through Facebook), it was the experience of the Stack Up community that their members needed a way to connect and receive support through video games.

In 2017, they implemented a new program within their veteran gaming community to provide mental health support and crisis intervention through their Discord server. This innovative program takes advantage of the anonymity, support, and connection of virtual communities based around shared experiences (military service, life stress) and interests (video gaming) to address gaps in the ability to provide support and connection to veteran and military gamers and others.

## Context

Like all mental health interventions, the program can be seen as operating within a variety of contexts. This section describes some of the organizational, technological, and cultural features in which the Overwatch Program is situated.

### Stack Up, a Veteran Serving Organization

Stack Up Founder and CEO Stephen “Shanghai Six” Machuga is a former Army Infantry/Military Intelligence officer and Airborne Ranger. Machuga says that video gaming “helped keep him sane” while he was deployed in Iraq with the 2nd Infantry Division and also played a significant role in his successful reintegration back into civilian life. Wanting the same for other veterans and active-duty military members, he founded Stack Up on Veterans Day, November 11, 2015.

Stack Up provides video game-related programs to support veterans and active-duty military globally. The Supply Crates and Stacks programs encourage in-person connection through co-located video game play, whereas the Air Assaults program promotes connectedness and community integration by giving selected veterans the opportunity to attend major video game and geek culture events with Stack Up leadership. Through veteran to veteran, veteran to civilian, and veteran to community connectedness, Stack Up helps veterans regain mental health and make successful transitions by providing programs that enable veterans to experience the therapeutic benefits of gaming and connections with others.

Stack Up also promotes an online community through its Discord server (see section “Discord and the Stack Up Server”). The community features several chat channels where veterans and others can connect through text or voice about games or any other topic. Weekly “Bored Room” meetings over voice chat keep members informed of the organization’s events and allow for regular member input and feedback. Most importantly, the server is home to Stack Up’s Overwatch Program (StOP), the first online crisis intervention program for veterans that is delivered entirely by trained volunteers through text and voice chat.

### Discord and the Stack Up Server

Discord is an online communication platform originally designed for the gaming community (Chiu and Mustafaraj, 2020) that has garnered 250 million users worldwide since its launch in 2015 (Discord software, 2020). Discord offers instant messaging, voice-over-Internet chat, and other computer-mediated communication and media sharing, allowing for extensive real-time social interaction between individuals and groups. Like other social media platforms, users can remain anonymous. While it has received some media attention, it has little mental health research compared with older platforms, such as Twitter, Facebook, and Reddit (Chiu and Mustafaraj, 2020).

The platform hosts the Stack Up server, a digital community of 2,727 members, offering many channels for live chat. Anyone may join Stack Up’s server; you do not need to be a veteran or even a gamer. Members use the channels to “hang out”—to talk in a lighthearted way about games and other common interests, to share memes and other visual media, and to relieve the stress of daily life during a pandemic, which now includes the traumatic stress from mandatory social isolation and fear of infection and death from coronavirus (Holingue et al., 2020; Holmes et al., 2020). These themes arise occasionally in the chat itself but are also reflected in requests for resources, information, and general help and support from veterans and other community members.

### Gaming Culture

The Stack Up community reflects many aspects of gaming and Internet culture. As a virtual community, they express their group identity through their public interactions around gaming (Grooten and Kowert, 2015). Members are known by their “handles,” usernames that identify them in the server and often in other social gaming spaces. Chat communication is filled with.GIFs (graphical interchange format ultra-short videos) and memes that emphasize, poke fun at, applaud, or comment on posts. During weekly voice chat member meetings, the #general channel fills with rapid-fire comments on Stack Up leadership’s (verbal) reports, emojis, or comments on those comments, GIFs, and memes in response, and so on. This rich, playful multimedia content fosters emotional connection and strengthens virtual community development (Hamilton et al., 2014).

### Crisis Intervention

Stack Up started its Discord server in 2015 and noticed early on that members would often voice their mental health needs in general channels that offered little to no privacy. Leadership at that time recognized that by offering crisis intervention directly, they might be able to reduce veteran suicide and promote good mental health in their virtual community. Once the Overwatch channel was started, many veterans offered to “lend an ear. Others shared their experiences, the ways they coped, and specific resources. As the program was formalized, training was developed in partnership with PsychArmor Institute, an organization providing training resources to help groups engage with and support veterans (PsychArmor, 2020). Volunteers take several modules through PsychArmor and then undergo hours of shadowing and supervised chat interaction prior to a scenario-based evaluation. Those who satisfactorily complete the training and interviews become official StOP team members, identified by the inclusion of [“StOP]” within their username. Team members need not be veterans or people with the lived experience of mental health problems, but contributions of StOP team members in the channel reflect the broader, non-VA definition of peer support as “mutual support delivered through sharing experiences of distress, difficulty and resilience” (Penney, 2018).

### Beginning the Public Health Evaluation Process

**FIGURE 1:**
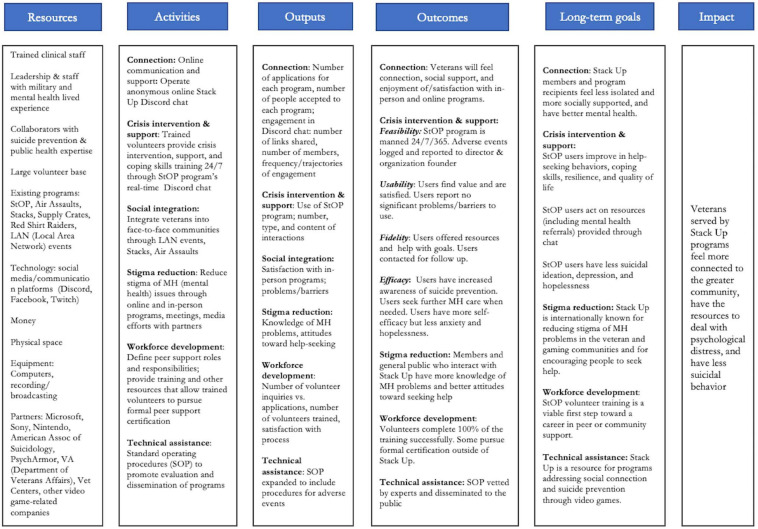
Stack Up logic model.

In 2018, Stack Up was selected as one of five veteran-serving organization recipients of the CDC (Centers for Disease Control and Prevention) *Veteran Suicide Prevention: Evaluation Demonstration Project*. The project team used the *CDC Evaluation Framework* (Milstein and Wetterhall, 1999) to build the infrastructure needed to perform evaluations of its programs. This formative evaluation focused on developing a logic model to describe the programs, conducting stakeholder engagement to identify evaluation goals and indicators, and identifying data sources needed to answer questions about how well the programs work. The logic model (Figure 1) was used to guide the current evaluation efforts.

## Essential Programmatic Elements

StOP is designed to meet basic standards for suicide prevention services including assessing suicide risk, promoting coping skills, and recommending follow-up. The program is delivered as a “peer-to-peer” intervention (see section “Conceptual and Methodological Constraints” for discussion of the peer support concept) and supervised by a licensed clinician.

### Staffing and Supervision

The StOP Team is led by author MB, a military veteran who obtained his graduate degree and clinical certification after his honorable discharge from the United States Air Force Security Services. Until June 2020, MB was the only paid staff member. He is supported by 25 volunteers, 15 of whom have completed training. StOP team members need not be veterans or have personal/lived experience with mental health problems or crisis. In some cases, individuals who have used StOP services have gone on to be trained to deliver the service. StOP also seeks to promote the development of a trained mental health support workforce and provide team members with valuable experience that could lead to paid work as a paraprofessional peer or community support specialist. So far, one StOP team member has secured paid employment as an online community manager with a large game development company.

The team communicates and solves problems through discussion in the #StOP-team-room chat channel, where MB maintains a constant presence during waking hours. The team is also able to seek advice from a clinical advisory board, which is usually consulted only when staff identify a possible duty to warn situation (the need to send emergency services to intervene in an imminent suicide attempt).

Unlike many other suicide prevention programs, Stack Up does not refer out—all contacts are handled directly by StOP team members, unless the user wishes support that is more specific to their needs. For example, users in the LGBTQ (lesbian, gay, bisexual, transgender and queer or questioning) community who want support from an LGBTQ-specific service may be referred to the Trevor project, a charity that focuses on suicide prevention for LGBTQ youth. Other suicide prevention initiatives, such as the Veteran Crisis Line, use multiple call centers, which may have differing practices or organizational structure that may make oversight complicated (Veterans Health Administration [VHA], 2019). However, StOP is still new. As it grows, it may need to expand to multiple groups or channels, which could reduce its ability to meet the needs of users exclusively from within StOP.

### Accessing the Service and Moving Between Channels

The Overwatch Program (StOP) has a public chatroom (#the-overwatch-program), private chatrooms devoted to training and team discussion, and public and private voice chats. Through these channels and chatrooms, it delivers crisis intervention and mental health support using trained volunteer peer supporters (StOP team members) to any member who requests to use it. StOP team members also maintain a presence in the #general channel, which allows them the opportunity to offer StOP support to anyone whose chat may indicate that they could use it.

**FIGURE 2:**
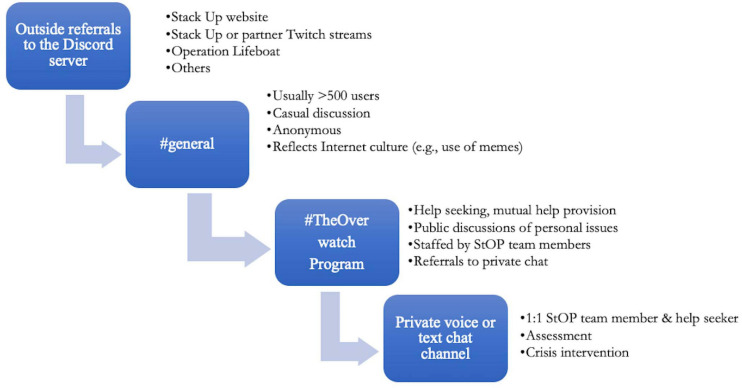
Stack Up Discord server chat and Stack Up Overwatch Program.

As shown in Figure 2, the service can be accessed through several different ways. Members can enter #the-overwatch-program channel directly and request help. If a member says something in the #general channel like “I’m really down; I just got fired and my VA benefits haven’t kicked in. I’m feeling really depressed,” another member or a StOP team member might suggest they post that in #the-overwatch-program. Users sometimes get referred from game streaming websites (e.g., Twitch) of Stack Up members or affiliates, and buttons and widgets on the Stack Up website link to #general and #the-overwatch-program. A feed from #the-overwatch-program is displayed on the website as a way to illustrate the nature of the chat, normalize help-seeking, and provide another anonymous way to get help. In June 2020, Stack Up began a unique partnership with wargaming.net’s World of Warships to offer Operation Lifeboat, a direct link to StOP from within the World of Warships digital game (colloquially known as “The Button”). This innovative intervention appears to be the first industry-supported bridge to mental health support from within a commercial video game designed for entertainment.

Although members at times seem to enjoy hanging out in #the-overwatch-program to talk about their issues and provide peer support, the channel as currently designed functions as a monitored public lobby where members are introduced to the team and program and receive general support. More specific crisis intervention is provided privately. Once a user has interacted with #the-overwatch-program, they or the StOP team member may request a private voice or text chat. These private chats follow best practices for crisis intervention as described in section “Defining and Responding to Crisis.” Members may request repeat private chats as needed, and StOP team members follow up with users in crisis.

### Defining and Responding to Crisis

Crisis intervention encounters are based on best practices and guided by the same formal rubric used to evaluate a trainee’s readiness to provide crisis intervention. Team members use a calm, welcoming, and non-judgmental approach, often using discussion of games to build rapport and explore issues. They are expected to have a broad knowledge of resources, many of which are contained in the StOP team member handbook or #training channel. In accordance with national standards for suicide risk assessment, StOP team members are encouraged to formally assess for suicidality by asking about current and recent suicidal ideation and then follow up with questions about suicidal plans, means, and past attempts if the user reports ideation (National Suicide Prevention Lifeline Standards, Training & Practices Subcommittee [NSPLSTPS], 2007). Other goals for the encounter are to discuss the life difficulties that brought the user to seek help, assess resiliency factors and support coping skills, offer resources (including referrals to mental health treatment), and co-develop an action plan. Team members collect information about the encounter and follow up within a few days of the contact. StOP emphasizes inclusivity and recently added specific training to help members meet the needs of the LGBTQ community.

One challenging standard for community-based crisis intervention services is the duty to warn situation. A duty to warn (also called duty to protect) situation occurs when a professional identifies a potential threat to life (the patient or someone else’s) and must break confidentiality to implement an intervention (e.g., calling police, notifying the family of someone in danger). Standards for suicide prevention services require that “Any program, which purports to be involved with life-threatening behaviors, must be, at the very least, capable of initiating or actually accomplishing a rescue in cases of life threatening acts already set in motion” (American Association of Suicidology [AAS], 2019, p. 57). Stack Up follows this standard by seeking informed consent from its users, assessing risk to life, seeking identifying information, and conducting rescue operations by contacting local emergency services when imminent risk is determined.

## Discussion

The Overwatch Program, a grassroots effort started by passionate volunteers, has evolved into an emerging model for providing mental health support through a focus on games and online connection. Although the program has been implemented in a large virtual community, from an evaluation standpoint is still in the early phases: Stack Up was just awarded a second grant by the CDC Foundation to conduct a formal evaluation of StOP and how it could be useful for veteran mental health, especially during the coronavirus disease 2019 (COVID-19) pandemic.

The evaluation will use interviews, a survey, and captured data to assess organizational factors, user characteristics, feasibility, usability, and efficacy (i.e., changes in processes or outcomes) of the program. We will also address critical questions related to the COVID-19 pandemic and mental health to get a picture of how this is impacting an online veteran community, such as the resources veteran members are able to access, including the VA care, and how lockdowns and other societal changes impact mental health and coping. The project uses a convergent/sequential design, mixed methods research approach to evaluate StOP and provide an initial scientific description of the Discord chat-based intervention. Our analysis is guided by the CDC Framework for Program Evaluation (Milstein and Wetterhall, 1999) as well as the World Health Organization guidance for evaluating digital health interventions (World Health Organization [WHO], 2016).

Although we have not yet collected enough data to inform an analysis, from previous data collection we know that 72 people used the StOP program last year, and so far this year, we have had 52 contacts. Interim data from the recently launched survey (*n* = 27 responses so far) show broad support for the program but also point to a few concerns. Some respondents appreciated knowing StOP was there if they needed it:

I think it’s appealing if someone is having a moment and need to vent especially dealing with PTSD [post-traumatic stress disorder] and depression.

I think it’s a great resource and just knowing it’s there is a great comfort. I think if we could get this to bases all around the country on the same level as other military sources like Military One Source, it could do even more good.

There are great people within it and I know that should I utilise the resource, I’d be speaking with those that actually care and can relate on some level.

One respondent pointed to the attributes of StOP team members as peers that they felt made the program valuable:

*Stack Up has mental health professionals on staff who are very up front with helping educate us and making sure that we are all doing ok. I think the biggest highlight of the StOP program is that all of the staff and volunteers do it because they care about and love every person that they help. Their interactions with the volunteers are on a very open and personal level, much more so than other professional services I’ve interacted with in the past*.

That said, we did find that some users expressed concerns about the program. Two respondents felt that there was a lack of specialty knowledge about the LGBTQ community, whereas one felt that training could be better. Several respondents felt that information about it was hard to find or was inadequate. These represent possible areas for improvement that are currently being addressed.

While our preliminary data do not reflect systematic analysis, asking open-ended questions is an evaluation approach that allows organizations to immediately address issues and improve program quality.

### Practical Implications

In the hopes that our description will be of use to other online communities, we provide here some lessons learned and practical implications for those who would like to translate this program to other virtual settings or conduct further research on StOP or Stack Up’s other programs.

#### Leverage the Skills and Passion of the Community

First, this program and its evaluation projects have required extensive time and commitment to launch, fund, and maintain. MB has been the passionate driver of the program for over 2 years, and his leadership has been invaluable for promoting the recognition of StOP in the suicide prevention community and securing funding for the program and its evaluation. MB brings his lived experience of being a veteran with PTSD to his professional leadership, informing and infusing the project with the values and preferences of those who have “been there.” As shown in the logic model, one of Stack Up’s major assets is the volunteers who are passionate about the Stack Up mission and the close-knit, grassroots community centered around video games and the military experience. Although StOP is based in a military gaming culture, it could easily translate to other online cultures. The tightknit nature seems critical to success, but communities can be built around other shared passions.

#### Incorporate Lived Experience

In contrast to forums or chatrooms designed as communities for mental health support, StOP provides a mental health intervention in a supportive existing community that was not originally designed to help users find mental health or social support. This is a novel model: StOP is created and led by an organization whose members have the lived experience of mental health challenges (like combat-related PTSD) and is delivered by trained members of the community rather than professionals. Use of community health workers to deliver mental health interventions is nothing new; such interventions provide an important way to shift tasks from professionals to paraprofessional community members in resource-constrained settings (Barnett et al., 2018).

One of Stack Up’s original goals for this program was to reduce veteran suicide by providing another avenue for veterans to seek mental health support and thus contribute to a suicide prevention mission, especially for veterans who are unable or unwilling to seek care from the VA. It also aims to provide an alternative to formal healthcare, as many veterans feel uncomfortable seeking help due to stigma or are unable to access it because resources are lacking (Castro, 2014; Kulesza et al., 2015). However, despite attending gaming conventions and suicide prevention conferences for years and being deeply embedded in the suicide prevention and “geek therapy” communities, we have seen no other examples of an online community—veteran oriented or not—that provides volunteer members with the combination of intensive training, supervision, and standards needed to deliver a suicide prevention intervention. This novel approach, combined with the potential for enormous reach, is what has brought StOP to the attention of the veteran suicide prevention community.

#### Disseminate New Knowledge and Practices

Social media monitoring and screening for suicide prevention is a hot topic for a research right now, and the Discord platform offers the opportunity for a new type of big data. The authors worked together to create a virtual suicide prevention conference (Operation HEAL: Suicide Prevention Conference-Home, 2018) to discuss how games and social media are used for suicide prevention^1^ and have participated in multiple panels together including at academic conferences (Colder Carras et al., 2018a, 2020b) and gaming conventions (Colder Carras et al., 2020a). The Stack Up community has spread information about its program through partnerships with streamers, social media suicide prevention communities, and industry. MB and MCC have discussed and promoted mental health support and crisis prevention through regular streaming efforts, including some focused on the COVID-19 pandemic and mental health (Stack Up, 2020). The gaming community has been extremely receptive and welcoming and has repaid Stack Up in volunteer time and fundraising efforts; last year, streamers raised over $250,000 for Stack Up programs.

#### Prepare for Research and Evaluation Early

Research activities have proven a challenge given the timeline of Stack Up’s fundraising efforts and travel, but the formative evaluation experience provided insight for organizational changes needed to sustain evaluation and research efforts. For a new or lean organization, time spent on evaluation may seem to compete with fundraising efforts. However, potential funders require data, so early investments in infrastructure pay off. It is vital to plan for resources, including staff time and support, to conduct program evaluation and research. Establish practices for evaluation and monitoring as you begin and start collecting data early to use in ongoing quality improvement.

New mental health support/crisis intervention programs should ensure not just that they are meeting the needs of community members, but that they do no harm. It is critical to have clear and intense training, testing, supervision, communication, and support for program volunteers. While all grassroots (i.e., community-developed) programs should have a thorough grounding in evidence-based practices, programs based in online communities also need to pay careful attention to privacy. Balancing confidentiality with ethical duty to intervene if suicide is imminent (American Association of Suicidology [AAS], 2019) is an imperative that new programs must be ready to address. This topic is developing in the research literature (see, e.g., Benton et al., 2017), but a greater review of these issues is beyond the scope of this paper.

#### Be Prepared for Change

Changes in technology or expansion to scale can have a powerful impact on programs. In support of the potential traffic from World of Warships’ 1 million players driven by Operation Lifeboat and “The Button,” StOP for the first time hired paid supervisory staff to cover the night shift. When communities transition from providing volunteer-based support to providing mixed volunteer and paid staff teams, this may affect how relationships are experienced and shift power dynamics within the team. Stack Up addressed this proactively for months as part of regular team meetings, but others wishing to use this model might consider having paid and volunteer staff from the outset so as to avoid the need for a cultural or attitudinal shift.

Stack Up also gained hundreds of new members within a week from this partnership, which pushed it from being a medium-sized to a large server on Discord. As a newly large server, the default ability to send push notifications for “at mentions[@name]” (e.g., “@Mat, I have a question”) was removed. StOP team members frequently use at mentions in the team channel to discuss real-time questions, so this feature is sorely missed, and work-arounds are currently being tested.

### Implications for Research and Policy

Creating mental health programs for and conducting mental health research within the gaming community are made much easier when these efforts are community- and peer-led. As members of mental health lived experience communities (i.e., people with the lived experience of mental health challenges) and gamers, MB and MCC bring in-depth, culturally informed perspectives to their joint projects that make planning easier and emphasize co-production of knowledge. Using professionals with lived experience to conduct research into mental health can be transformative for mental health systems and services (Jones et al., 2014), and transformation and innovation are needed for both suicide prevention and veteran mental healthcare (National Institute of Mental Health [NIMH], 2019). Lived experience research leadership streamlines the incorporation of multiple perspectives and can help ensure a focus on community values, preferences, and cultural considerations.

The setting of an online gaming community and a chat-based platform is extremely well-positioned to support program implementation and research. Text chat can be anonymous, allowing people with mental health needs to seek support privately, and the setting of a mental health promoting, stigma-busting community normalizes help seeking. Synchronous and asynchronous chats in the StOP team room foster connection between team members but also allow for rapid response and supervision/intervision (peer problem solving).

The combination of military and gaming communities also challenges existing approaches to social media data analysis. For example, use of vulgar language (swear words) has been considered a sign of negative sentiment in some approaches to social media data analysis, but could also reflect group identity or signal informal conversation (Cachola et al., 2018). For male veteran gamers, “trash talking” may be an integral part of the gaming experience (Colder Carras et al., 2018a). That said, natural language processing and other machine learning methods used in suicide prevention (e.g., Coppersmith et al., 2018) will be a great next step, especially as links between suicide and risk factors, such as alexithymia (difficulty identifying and describing emotions in oneself and others; De Berardis et al., 2017), are better understood.

We have been approached by several non-gaming organizations seeking to implement similar programs and can highly recommend that interested researchers or program directors immerse themselves in the communities they seek to serve or work with. There is no substitute for being there, day after day, week after week, offering attention and appreciation while being mindful of the limits of one’s own understanding.

### Conceptual and Methodological Constraints

In describing StOP, we face several challenges and methodological constraints. Meetings with potential funders can be challenging as the authors must bridge many knowledge gaps related to online communications, the Discord platform and the privacy implications of public discussions of mental health and suicide. The Stack Up community is itself hard to describe—it is not limited to a specific game or to veterans, but made up of people who play multiple games (or do not game much) as well as veterans, military service members, and citizens.

Another challenge is understanding the meaning of peer-to-peer support in StOP. Stack Up uses that term to describe an intervention that uses community health workers, e.g., “interventionists without formal mental health training and who are members of the community they serve” (Barnett et al., 2018). This is in contrast to the way it is often seen in the VA and other mental health service settings in the United States, where the term “peer” in peer support implies having the lived experience of the mental or behavioral health condition one is supporting. Importantly, Stack Up does not require team members to have lived experience, but it does support and encourage people who are veterans or who have lived experience to apply.

One big challenge for understanding online Discord-based communities from a public health perspective is defining what it means to be a member of the population. In public health, we aim to define populations from which samples and study participants can be drawn. We think of the Stack Up server as having a population of over 2,700 members, but this fluctuates from month to month. The state of being a member is relatively absorbent—once you become a member, you stay a member, even if you no longer participate in the server. The only way to change this is to quit (or be banned, which is not uncommon in some online communities). Some online communities reflect the approaches used in marketing and online media, measuring activity and membership as active or not based on various online behaviors (posting, logging in, etc.). This poses a challenge to data collection and inference, because scientists will need to develop new ways to think about populations and who can be considered in the sample of those who may benefit (or be put at risk by) online activities and groups.

Evaluating mental health programs for gaming or other online communities requires not just a public mental health lens but also an informatics lens and ample researcher reflexivity. Grassroots organizations can have tremendous success at developing and implementing programs but be less prepared for the steps required for a rigorous evaluation. It is vital to have a clear understanding of what is needed to conduct proper evaluation research and ongoing monitoring to ensure that the community’s mental health is supported and research participants’ privacy is protected.

## Conclusion

As far as we know, this is the first description of a formal, volunteer-driven crisis intervention program in an online gaming setting. By taking advantage of new computing approaches, using data from outside healthcare settings in health research, applying algorithms and data analytics to assess the value and efficacy of interventions, and describing an intervention designed to connect veterans with each other and the community at large, the current evaluation project addresses several strategies and goals of the NIMH Strategic Plan (National Institute of Mental Health [NIMH], 2020) as well as the Executive Order to End Veteran Suicide (EO13861). Using a public chatroom to deliver crisis intervention services is a potentially disruptive form of suicide prevention—while many interventions exist to identify potential suicidality in social media postings (e.g., Facebook’s feature that allows users to report other users’ posts), such interventions provoke the ethical dilemma of what to do when an online risk analysis identifies an individual at need of crisis intervention in real time (National Institute of Mental Health; Coppersmith et al., 2018). The intervention presents challenges in terms of understanding what it means to be a community member and how it fits within the models of peer- or community-based support, but by outlining these and the potential benefits, we are hopeful that other researchers and organizations can learn from this information and leverage the power and passion of gaming communities to promote mental health and prevent suicide.

## Data Availability Statement

The datasets presented in this article are not readily available because minimal preliminary data is presented here. The full dataset will be made available when the study is complete. Requests to access the datasets should be directed to MCC, mcarras@jhu.edu.

## Ethics Statement

The studies involving human participants were reviewed and approved by the Institutional Review Board of the Johns Hopkins Bloomberg School of Public Health. Written informed consent for participation was not required for this study in accordance with national legislation and institutional requirements.

## Author Contributions

MCC and AL: conceptualization. MCC and MB: funding acquisition. MCC: investigation, project administration, and writing—original draft. MCC, MB, and AL: methodology, writing—review, and editing. All authors contributed to the article and approved the submitted version.

## Conflict of Interest

MCC is currently seeking funding from industry sources to conduct game research. She serves on the Stack Up advisory board and is the CEO and founder of the Gaming and Wellness Association, Inc., a non-profit video game research organization. MB is the Director of Suicide Prevention at Stack Up. The remaining author declares that the research was conducted in the absence of any commercial or financial relationships that could be construed as a potential conflict of interest.

## Footnotes

1 https://tinyurl.com/OpHealConf
